# rMAP-Candida: a modular Dockerized WDL/Cromwell workflow for reproducible *Candida species* typing, assembly-contiguity assessment, antifungal-resistance marker screening, and phylogenomic surveillance

**DOI:** 10.3389/fbinf.2026.1896572

**Published:** 2026-08-12

**Authors:** Gerald Mboowa, Ivan Sserwadda, Stephen Kanyerezi, Benson R. Kidenya, Jonani Bwambale, Benson Musinguzi

**Affiliations:** 1 The African Centre of Excellence in Bioinformatics and Data-Intensive Sciences, Makerere University, Kampala, Uganda; 2 Department of Immunology and Molecular Biology, School of Biomedical Sciences, College of Health Sciences, Makerere University, Kampala, Uganda; 3 Department of Biochemistry and Molecular Biology, Weill Bugando School of Medicine, Catholic University of Health and Allied Sciences, Mwanza, Tanzania; 4 African Bioinformatics Institute (ABI), Cape Town, South Africa; 5 Department of Medical Laboratory Sciences, Faculty of Health Sciences, Muni University, Arua, Uganda

**Keywords:** antifungal resistance, bioinformatics workflow, *Candida* spp., fungal genomics, genomic surveillance, rMAP-Candida

## Abstract

**Background:**

*Candida* spp. infections are an increasing public health concern, particularly in settings where laboratory mycology, genomic surveillance infrastructure, and antifungal susceptibility testing remain limited. Accurate species identification, reproducible assembly assessment, conservative genomic screening for antifungal-resistance markers, and interpretable phylogenomic outputs are essential for surveillance and outbreak preparedness. However, fungal whole-genome sequencing workflows remain fragmented, difficult to reproduce across computing environments, and insufficiently adapted for implementation in low-resource public health genomics settings.

**Methods:**

We developed rMAP-Candida, a modular, Dockerized WDL/Cromwell workflow for paired-end *Candida* spp. whole-genome sequencing analysis. The workflow performs read quality control and trimming with fastp, Candida-focused species typing using Kraken2/Bracken, *de novo* assembly with MEGAHIT, assembly-contiguity assessment with QUAST, optional genome-completeness assessment using Compleasm or BUSCO, antifungal-resistance marker screening using ChroQueTas/FungAMR-derived outputs, species-aware core-SNP phylogenomics, pairwise SNP-distance summarization, closest-neighbor analysis, and integrated HTML surveillance reporting. To improve independent reproducibility, the repository includes a quick-start local Cromwell test, a corrected two-sample input JSON, documented checks for public container and database access, and a two-sample reproducibility run.

**Results:**

rMAP-Candida generated reproducible species assignments, assembly-contiguity metrics, optional completeness summaries, antifungal-resistance marker outputs, species-aware phylogenomic summaries, pairwise SNP-distance tables, closest-neighbor summaries, and integrated HTML reports. In the Ugandan validation dataset, the workflow identified six principal species groups, dominated by *Candida albicans*, followed by *Candida tropicalis*, *Pichia kudriavzevii*, *Nakaseomyces glabratus*, *Clavispora lusitaniae*, and *Candida parapsilosis*. The integrated report summarized 24 antifungal-resistance marker hits and a median assembly N50 of 35,946 bp. Species-aware phylogenomics was performed for eligible species groups, while ineligible or skipped groups were explicitly reported with reasons. The report also distinguished “no curated genomic antifungal-resistance marker detected” from phenotypic susceptibility, supporting conservative interpretation of resistance-screening outputs.

**Conclusion:**

rMAP-Candida provides a portable, reproducible, modular, and surveillance-oriented WDL/Cromwell workflow for *Candida* spp. genomic analysis. By integrating species identification, assembly-contiguity assessment, optional completeness evaluation, antifungal-resistance marker screening, species-aware phylogenomics, SNP-distance summarization, closest-neighbor reporting, and HTML reporting, the workflow supports training, research, and applied fungal genomic surveillance in low-resource and other implementation settings.

## Introduction

*Candida* spp. are important causes of mucosal and invasive fungal disease, with candidemia and invasive candidiasis associated with substantial morbidity, mortality, prolonged hospitalization, and health-system burden (Bays et al., 2024; Denning, 2024). Recent global estimates suggest that approximately 1.56 million people develop *Candida* spp. bloodstream infection or invasive candidiasis annually, resulting in nearly 1 million deaths, with crude mortality rates exceeding 60% in many settings (Denning, 2024). The emergence and international spread of multidrug-resistant *Candidozyma auris*, commonly referred to clinically as *Candida auris*, has intensified the need for timely, reproducible, and scalable fungal genomic surveillance (World Health Organization, 2022; Chow et al., 2020; Lockhart et al., 2017). Whole-genome sequencing can support species identification or confirmation, detection of unexpected species assignments or possible mixed-species signals, outbreak investigation, phylogenomic comparison, and screening for resistance-associated genetic markers (Chow et al., 2020; Lockhart et al., 2017; Berman and Krysan, 2020). However, in many implementation settings, fungal genomic workflows remain difficult to deploy because they require multiple standalone tools, complex software environments, curated reference databases, species-specific analytical decisions, and careful interpretation of species-identification, assembly-quality, phylogenomic, and antifungal-resistance marker outputs (Berman and Krysan, 2020; Chaabane et al., 2019; Pristov and Ghannoum, 2019).

These implementation challenges are particularly important in Africa, where fungal disease surveillance, diagnostic mycology infrastructure, antifungal susceptibility testing, and access to specialized bioinformatics expertise remain limited, despite the World Health Organization’s 2022 publication of the first fungal priority pathogens list, which classified 19 fungal entities into critical-, high-, and medium-priority groups (World Health Organization, 2022; Casalini et al., 2024). Therefore, a practical genomic surveillance workflow that is reproducible, portable, transparent, modular, and capable of generating outputs that are interpretable by both bioinformaticians and public health laboratory teams is urgently needed to strengthen fungal disease surveillance, support timely detection of emerging resistance, and improve outbreak preparedness (Wratten et al., 2021; Ahmed et al., 2021; Voss et al., 2017). These challenges are not only infrastructural but also methodological, particularly in the interpretation of genomic antifungal resistance signals generated through sequencing workflows. Given these limitations, interpretation of antifungal resistance based solely on genomic marker detection requires caution. In particular, genomic marker detection should be reported as screening evidence rather than as a definitive clinical susceptibility result, because resistance phenotypes may depend on species-specific mechanisms, incomplete marker catalogues, copy-number variation, aneuploidy, loss of heterozygosity, regulatory changes, and local epidemiological context (Berman and Krysan, 2020; Pristov and Ghannoum, 2019; Alastruey-Izquierdo and Martín-Galiano, 2023). Where available, genomic findings should therefore be interpreted alongside validated phenotypic antifungal susceptibility testing and clinical laboratory review (Berman and Krysan, 2020; Pristov and Ghannoum, 2019; Alastruey-Izquierdo and Martín-Galiano, 2023).

To address this gap, we developed rMAP-Candida, a rapid mycological analysis pipeline for paired-end *Candida* species whole-genome sequencing data. The workflow is implemented in Workflow Description Language (WDL), executed using the Cromwell workflow engine, and containerized with Docker to standardize software dependencies across laptops, workstations, high-performance computing (HPC) environments, and cloud-compatible execution platforms (Wratten et al., 2021; Ahmed et al., 2021; Voss et al., 2017; Boettiger, 2015; Merkel, 2014). This design allows the same workflow to be run on a researcher’s laptop for small test datasets, on institutional HPC infrastructure for larger analyses, or in cloud environments for scalable and reproducible genomic surveillance.

rMAP-Candida integrates a modular set of established bioinformatics tools to ensure reproducibility and transparency at each analytical stage. Read-level quality control and adapter trimming are performed using fastp (Chen et al., 2018), with optional quality reporting through FastQC (Babraham Bioinformatics, 2026) and MultiQC (Ewels et al., 2016). Species identification is conducted using Kraken2 (Wood et al., 2019) with abundance refinement using Bracken (Lu et al., 2017), enabling *Candida* spp.-focused taxonomic resolution. *De novo* genome assembly is performed using MEGAHIT (Li et al., 2015), followed by assembly quality evaluation using QUAST (Gurevich et al., 2013) to assess contiguity metrics including N50, contig count, and assembly length. Optional gene-level genome completeness assessment can be done using Compleasm (Huang and Li, 2023) or BUSCO (Simão et al., 2015) when deeper completeness evaluation is required.

The workflow also performs antifungal-resistance marker screening using ChroQueTas/FungAMR-derived outputs, species-aware core-SNP phylogenomics, maximum-likelihood tree inference and rendering, pairwise SNP-distance summarization, closest-neighbor reporting, and generation of an integrated HTML surveillance report (Wratten et al., 2021; Huerta-Cepas et al., 2016; Bédard et al., 2025; Minh et al., 2020; Seemann, 2026). In the current implementation, phylogenomic analysis is performed in a species-aware manner, where eligible species groups are processed separately using species-appropriate NCBI RefSeq reference genomes and configurable phylogeny branches, including Snippy-based variant calling and core-SNP alignment (Seemann, 2026) for *Candidozyma auris* and *Candida albicans*, with support for additional configured species groups where appropriate. Species groups that are not eligible for phylogeny because of insufficient sample numbers, unavailable reference configuration, or failed intermediate outputs are explicitly reported rather than silently excluded, supporting transparent interpretation of workflow outputs.

**FIGURE 1:**
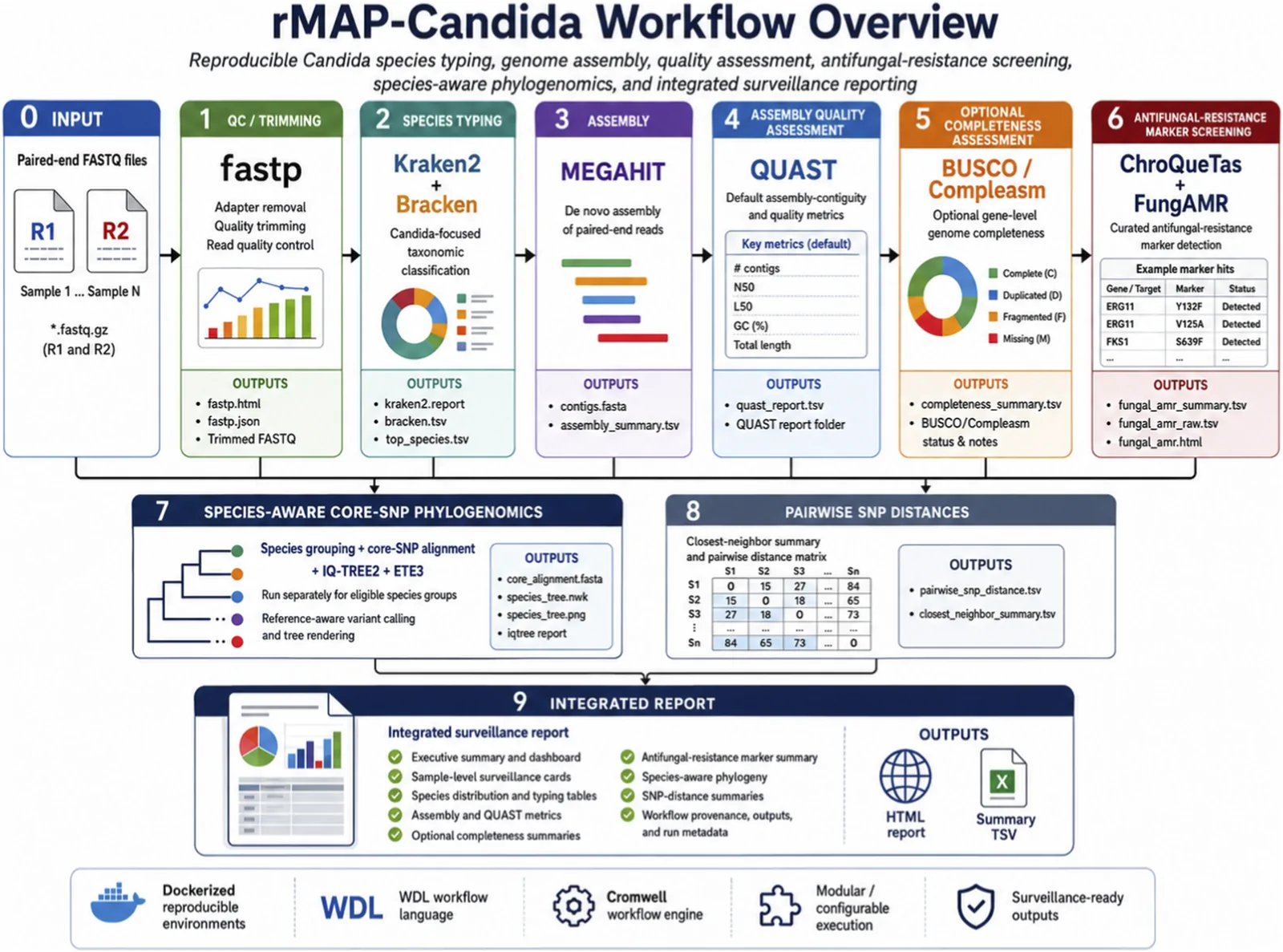
Overview of the rMAP-*Candida* workflow. The workflow accepts paired-end FASTQ files as input and performs: (1) read trimming and quality control using fastp; (2) *Candida*-focused species typing using Kraken2 and Bracken; (3) *de novo* genome assembly using MEGAHIT; (4) default assembly-contiguity and quality assessment using QUAST; (5) optional genome completeness assessment using BUSCO or Compleasm; (6) antifungal-resistance marker screening using ChroQueTas and FungAMR-derived outputs; (7) species-aware core-SNP phylogenomics, including species grouping, reference-aware variant calling, core-SNP alignment, maximum-likelihood tree inference, and tree rendering; and (8) pairwise SNP-distance and closest-neighbor summarization. Outputs from all workflow modules are integrated into a surveillance-oriented HTML report and summary tables, including executive summaries, sample-level cards, species distribution, assembly metrics, AMR marker summaries, phylogenomic outputs, SNP-distance tables, workflow provenance, and run metadata. The workflow is Dockerized, implemented in Workflow Description Language (WDL), and executed using the Cromwell workflow engine to support reproducibility, portability, and modular deployment across local and cloud-based environments. Abbreviations: QC, quality control; WDL, Workflow Description Language; BUSCO, Benchmarking Universal Single-Copy Orthologs; AMR, antimicrobial/antifungal resistance.

Here, we describe the design, validation strategy, and benchmark performance of rMAP-Candida across public *Candida* spp. datasets, including a 34-isolate Ugandan clinical *Candida* spp. validation dataset. The main contribution of rMAP-Candida is the operational integration of established genomics tools into a single reproducible, modular, and surveillance-oriented workflow. Rather than introducing a new species classifier, assembler, or phylogenetic method, rMAP-Candida standardizes the execution, quality assessment, summarization, interpretation, and reporting of these components for applied *Candida* spp. genomic surveillance. The workflow emphasizes portability across local, HPC, and cloud environments; reproducibility through Dockerized WDL/Cromwell execution; modularity through user-defined switches; conservative interpretation of genomic antifungal-resistance markers; and public-health usability through integrated HTML and tabular outputs. The overall workflow structure, from paired-end FASTQ input through integrated HTML reporting, is summarized in Figure 1.

## Methods

### Workflow design and implementation

rMAP-Candida was designed as a modular WDL/Cromwell workflow for paired-end Illumina *Candida* spp. whole-genome sequencing FASTQ data. The workflow accepts matched sample names, read 1 FASTQ files, and read 2 FASTQ files as core inputs, with optional surveillance metadata for downstream interpretation in the integrated report. User-configurable Boolean switches allow individual modules to be enabled or disabled, including read trimming, read-level quality control, species typing, *de novo* assembly, QUAST-based assembly-contiguity assessment, optional gene-level genome completeness assessment using Compleasm or BUSCO, antifungal-resistance marker screening, species-aware phylogenomics, and phylogenetic tree rendering. In the default rapid surveillance configuration, QUAST is used to provide assembly-contiguity and quality metrics, while Compleasm and BUSCO remain optional modules that can be enabled when gene-level completeness assessment is required.

The stable workflow uses Dockerized tasks (Table 1) to support reproducible execution across local, HPC, and cloud-compatible WDL platforms. Key outputs include per-sample read-quality summaries, optional MultiQC reports, species-typing tables, assembled contigs, QUAST assembly metrics, optional Compleasm/BUSCO completeness summaries, antifungal-resistance marker summaries, species-aware phylogeny group summaries, core-SNP alignments, Newick trees, IQ-TREE reports, rendered tree images, pairwise SNP-distance tables, surveillance summary tables, and an integrated HTML report.

**TABLE 1:** Overview of the rMAP-Candida workflow architecture, software provenance, and key outputs.

Module	Tool/method	Version/provenance used	Purpose	Key outputs
Read QC and trimming	fastp	fastp v1.3.6	Adapter and quality trimming; read-level QC	fastp HTML/JSON summaries; trimmed FASTQ files
Optional read QC summary	FastQC; MultiQC	FastQC v0.12.1; MultiQC v1.35	Optional per-sample and aggregate read-quality reporting	FastQC HTML/ZIP outputs; MultiQC HTML report
Species typing	Kraken2; Bracken	Kraken2 v2.17.1; Bracken v3.1p1; Candida-focused database image gmboowa/rmap-myc-candida-kraken2-bracken:2026.05-db	Candida-focused taxonomic classification and species-level abundance estimation	Kraken2 report; Bracken species table; top-species TSV
Assembly	MEGAHIT	MEGAHIT v1.2.9	*De novo* assembly of paired-end reads	Contigs FASTA; assembly summary TSV
Assembly QC	QUAST	QUAST v5.3.0	Assembly-contiguity and GC-content metrics	Transposed QUAST report TSV; QUAST report directory; N50; contig count; total length; GC content
Optional completeness assessment	BUSCO; Compleasm	BUSCO v6.1.0; Compleasm v0.2.8	Optional gene-level genome completeness assessment using fungal/Candida-appropriate lineage settings	BUSCO short summary; BUSCO full table; BUSCO output directory; Compleasm summary; Compleasm full table; lineage-specific output directory
Antifungal-resistance marker screening	ChroQueTas/FungAMR-derived output parsing	rMAP-Candida AMR container gmboowa/rmap-myc-candida-amr:2026.07-chroquetas-v9; Dockerfile and container validation test documented under docker/amr_chroquetas/	Curated marker-level genomic antifungal-resistance screening	Fungal AMR summary TSV; raw marker TSV; AMR HTML report
Species-aware phylogenomics	Snippy/core-SNP alignment; IQ-TREE; ETE3	Snippy v4.6.0; IQ-TREE v3.1.3; ETE3 v3.1.3; reference container gmboowa/rmap-candida-refs:2026.05	Species-specific core-SNP phylogenomic analysis and visual tree rendering	Core-SNP alignment; Newick tree; rendered PNG tree; IQ-TREE report
SNP-distance summary	Built-in report parser	rMAP-Candida workflow release/Git commit and Docker image tags recorded in run metadata	Pairwise SNP-distance and closest-neighbor summarization for genomic epidemiology interpretation	Pairwise SNP-distance TSV; closest-neighbor summary
Reporting	Built-in reporting task	rMAP-Candida workflow release/Git commit, and reporting Docker image tag recorded in run metadata; Cromwell 91 with the supplied Local/SFS configuration for validated local Docker/Colima execution	Integrated surveillance-oriented HTML report	rMAP_*Candida*_report.html; rMAP_*Candida*_summary.tsv; surveillance summary TSV

User-configurable Boolean switches enable or disable each major module. The version/provenance column records the software versions used, together with the custom Docker/database tags that are pinned in WDL, inputs and captured in run metadata.

Repository-level reproducibility was strengthened by providing a quick-start route that runs the workflow locally using Cromwell 91 together with the supplied example/cromwell.local.fifo_portable.conf Local/Shared File System configuration. The example input JSON uses two public test samples, ERR263534 and ERR331060, and provides a minimal reproducibility run for verifying the expected HTML and TSV outputs.

The workflow supports configurable execution through true/false switches in the input JSON, allowing optional components such as completeness assessment, antifungal-resistance marker screening, phylogenomic analysis, and reporting steps to be enabled or disabled without modifying the WDL. This preserves the tested single-WDL execution model while supporting modular use, transparent configuration, and reproducible execution across computing environments.

### Validation datasets

rMAP-Candida was evaluated using *Candida* spp. whole-genome sequencing datasets of different sizes and sources. Publicly available SRA/NCBI datasets were selected to represent clinically relevant Candida-related species, known or expected species assignments, and resistance-marker-positive controls where available. These were organized into small, medium, and large validation batches of 17, 25, and 50 samples, respectively, to assess workflow functionality, scalability, and report generation across increasing sample numbers.

Together, the public benchmark datasets and the Ugandan clinical dataset enabled evaluation of species typing, assembly-contiguity assessment, optional completeness assessment, antifungal-resistance marker screening, species-aware phylogenomics, SNP-distance summarization, and integrated HTML reporting across both benchmark and local surveillance contexts. The validation datasets, sample numbers, data sources, intended purpose, and main outputs checked are summarized in Table 2. In addition to the larger validation batches, a minimal two-sample test was added to the repository to confirm installation-free reproducibility. This test uses ERR263534 and ERR331060 and was run locally using Cromwell 91 together with the supplied example/cromwell.local.fifo_portable.conf Local/SFS configuration, as documented in the Software availability section. The configuration supports reliable Docker/Colima execution on local machines by defining task concurrency, Docker resource mapping, container-visible task scripts, and native/tmp FIFO handling.

**TABLE 2:** Validation and demonstration datasets used to evaluate rMAP-*Candida*.

Dataset	Number of samples	Source	Evaluation purpose	Main outputs checked
Small public validation batch	17	Public SRA/NCBI *Candida* reads	Workflow functionality	QC, species typing, assembly, report generation
Medium public validation batch	25	Public SRA/NCBI *Candida* reads	Multi-sample reporting	Species distribution, AMR summary, assembly metrics
Large public validation batch	50	Public SRA/NCBI *Candida* reads	Scalability testing	Runtime, sharding, report stability
Uganda clinical validation batch	34	Ugandan clinical *Candida* reads available from NCBI/SRA	Local surveillance relevance	Species typing, AMR marker screening, assembly QC, species-aware phylogenomics

AMR, antimicrobial/antifungal resistance; QC, quality control.

### Interpretation of antifungal-resistance outputs

Antifungal-resistance outputs were interpreted as genomic screening evidence rather than as direct phenotypic susceptibility calls. The workflow reports curated marker-level evidence where available, including the affected gene, detected mutation, associated antifungal drug class, evidence level, and interpretation field. Importantly, samples without detected curated markers are reported as “No curated genomic AMR marker detected: susceptibility not inferred,” because absence of a marker in the configured database does not establish phenotypic susceptibility.

This conservative interpretation was intended to reduce overstatement of genomic AMR findings. *Candida* spp. antifungal resistance can involve diverse and species-specific mechanisms, including point mutations, promoter alterations, copy-number variation, aneuploidy, loss of heterozygosity, efflux regulation, and other regulatory or structural changes that may not be fully captured by short-read marker screening or by the configured resistance catalogue. Therefore, rMAP-Candida outputs should be interpreted as surveillance and research evidence, ideally alongside species identity, sample metadata, validated mutation catalogues, and phenotypic antifungal susceptibility testing where available.

The custom *Candida* spp.-focused Kraken2/Bracken database is distributed through the public Docker image gmboowa/rmap-myc-candida-kraken2-bracken:2026.05-db, with the database located at/opt/kraken2_db/*candida* inside the container. The repository includes commands to pull the image, list the database files, and verify the expected Kraken2 database components. This ensures that species-typing resources are publicly accessible and auditable.

### Species-aware phylogenomics

For phylogenomic analysis, rMAP-Candida first groups isolates by species before variant calling, core-SNP alignment, and tree construction. This species-aware design avoids combining genetically divergent *Candida* species into a single core-SNP phylogeny, which could lead to inappropriate comparisons and misleading genomic-distance interpretation.

For each eligible species group, the workflow requires a minimum number of same-species samples and a configured species-matched reference genome, currently selected from NCBI RefSeq reference genomes where available. Eligible groups are processed through species-specific variant-calling branches, followed by core-site filtering, maximum-likelihood tree inference, tree rendering, pairwise SNP-distance calculation, and closest-neighbor summarization. Species groups with too few samples, missing reference configuration, or insufficient successful consensus outputs are explicitly reported as skipped rather than silently excluded, improving transparency and helping users distinguish analytical ineligibility from true absence of phylogenomic signal.

This conservative, species-aware design also addresses *Candida* genome biology. *Candida* and related yeasts differ in ploidy, heterozygosity, genome plasticity, aneuploidy, copy-number variation, loss of heterozygosity, and structural variation (Oggenfuss et al., 2025; Vande et al., 2023; Todd et al., 2019; Selmecki et al., 2010). Therefore, rMAP-Candida does not assume that a single haploid, pan-Candida SNP model is appropriate for all species. Instead, it separates species before phylogenomics, applies configurable species-specific branches, reports skipped or ineligible groups explicitly, and interprets SNP distances as descriptive genomic relatedness metrics rather than definitive transmission evidence.

## Results

### Pipeline outputs and reporting

**FIGURE 2:**
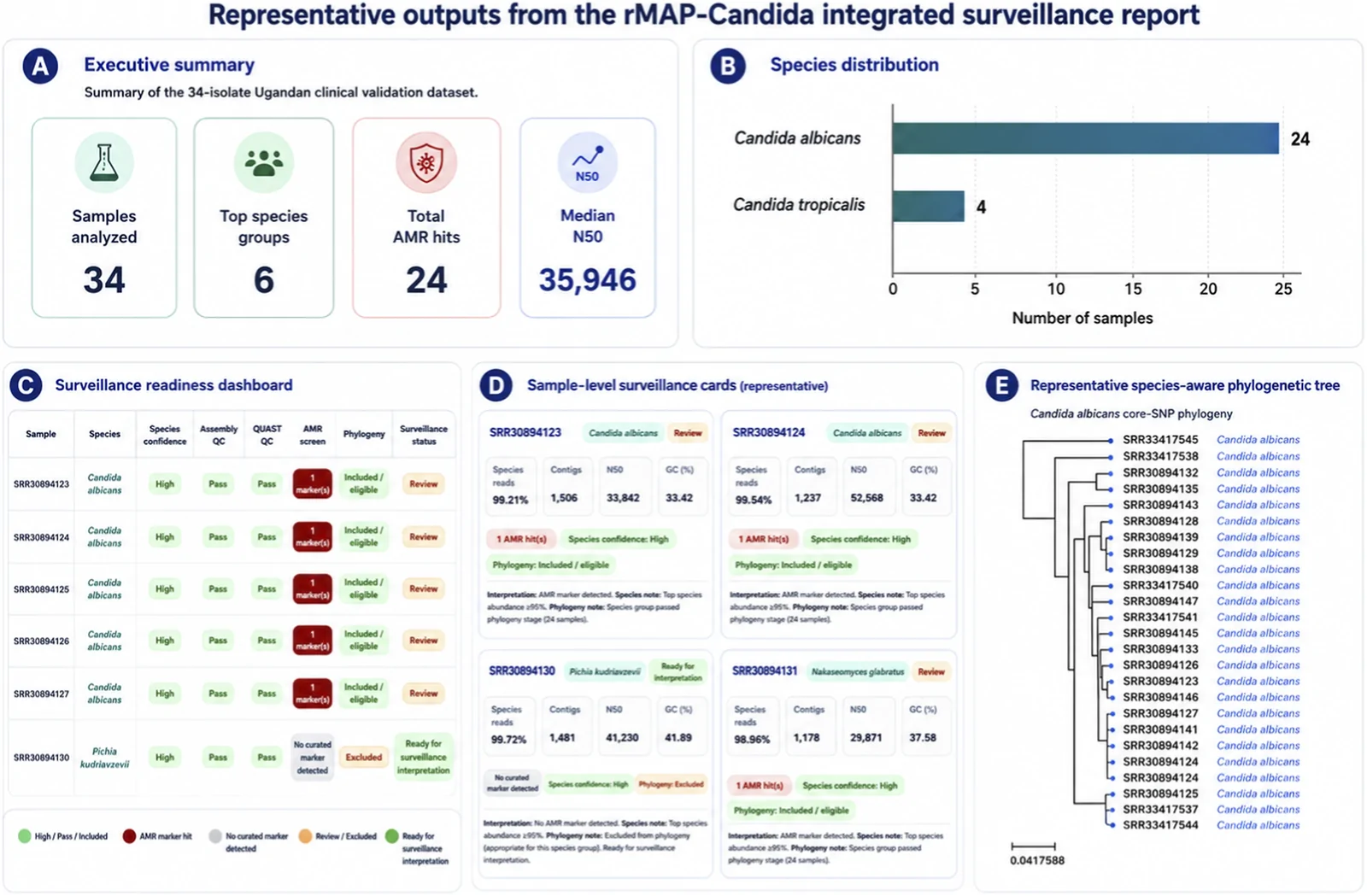
Representative extract from the integrated rMAP-Candida HTML surveillance report. The figure shows selected components of the report generated from the 34-isolate Ugandan clinical validation dataset, including: **(A)** executive summary metrics; **(B)** distribution of the two most frequent species groups; **(C)** representative surveillance-readiness dashboard rows; **(D)** example sample-level surveillance cards; and **(E)** a representative species-aware core-SNP phylogenetic tree. The full HTML report contains additional samples, complete species-typing tables, assembly-contiguity and QUAST metrics, antifungal-resistance marker summaries, phylogeny eligibility information, SNP-distance summaries, interpretation notes, and workflow provenance, and is available at: https://gmboowa.github.io/rMAP-Candida/reports/Ugandan_dataset/report.html. The report is designed to support transparent review of species identity, assembly quality, genomic antifungal-resistance screening, phylogenomic inclusion status, and surveillance-readiness outputs.

The integrated HTML report is designed for surveillance interpretation and includes an executive summary, sample-level cards, species distribution summaries, Kraken2/Bracken species typing tables, MEGAHIT assembly summaries, QUAST assembly-quality tables, optional Compleasm or BUSCO completeness outputs, fungal AMR marker tables, species-aware phylogeny summaries, rendered tree images, pairwise SNP-distance and closest-neighbor tables, downloadable output links, and run metadata. A representative extract from the integrated HTML surveillance report generated from the Ugandan clinical validation dataset is shown in Figure 2.

The reporting module emphasizes interpretability and transparency by distinguishing successful outputs from skipped or unavailable analyses. For example, species groups that do not meet phylogeny requirements because of insufficient sample numbers, missing reference configuration, or failed intermediate outputs are reported as skipped rather than omitted. Similarly, antifungal-resistance results distinguish detected curated markers from samples in which no curated genomic AMR marker was identified, avoiding interpretation of marker absence as phenotypic susceptibility.

### Two-sample reproducibility run

The repository includes a two-sample reproducibility run using ERR263534 and ERR331060. This run was executed locally using Cromwell 91 together with the supplied example/cromwell.local.fifo_portable.conf Local/SFS configuration. The integrated report summarized two analyzed samples, two top species groups, one curated AMR marker hit, and a median assembly N50 of 61,358 bp. ERR263534 was assigned to *Candida albicans* with high species confidence and one curated AMR marker hit, while ERR331060 was assigned to *Nakaseomyces glabratus* with a low-confidence species assignment requiring review. Species-aware phylogeny was correctly skipped because each species had fewer than the default minimum of three same-species samples.

### Representative sample validation report

**FIGURE 3:**
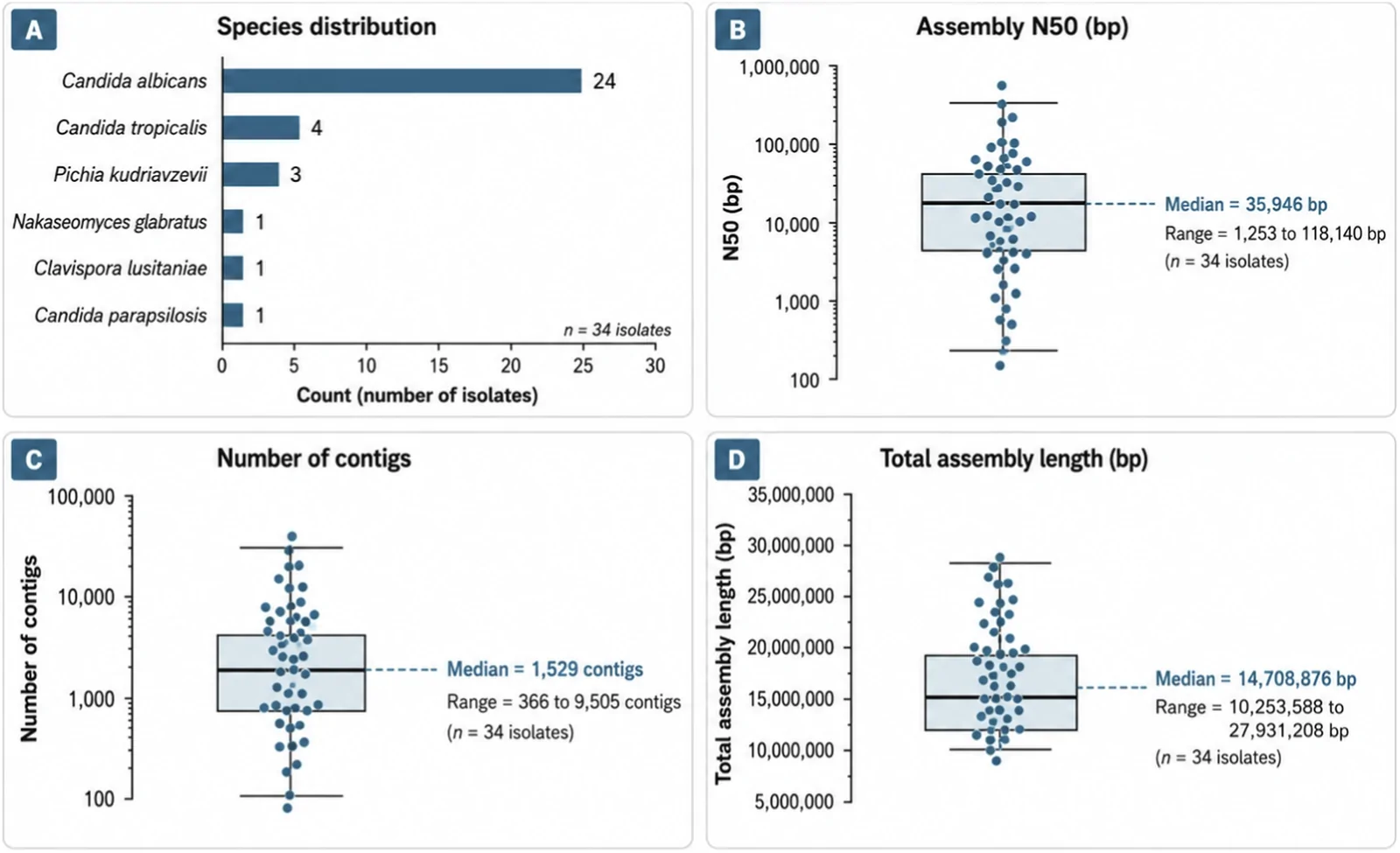
Species distribution and assembly-quality summary for the 34-isolate Ugandan clinical *Candida* spp. validation dataset. **(A)** shows the distribution of species groups identified across the dataset, with *Candida albicans* representing the dominant species group. **(B–D)** summarize QUAST-derived assembly-contiguity metrics across all 34 isolates, including assembly N50, number of contigs, and total assembly length. Boxplots show the median and interquartile range, with individual points representing sample-level assemblies. The median assembly N50 was 35,946 bp, the median number of contigs was 1,529, and the median total assembly length was 14,708,876 bp. These results provide an overview of species composition and assembly quality for evaluating rMAP-Candida performance on a Uganda-linked clinical validation dataset.

In the 34-isolate Ugandan clinical validation report, rMAP-Candida analyzed 34 paired-end *Candida* spp. genome datasets and detected six top species groups. The dominant species group was *Candida albicans* with 24 isolates, followed by *Candida tropicalis*, *Pichia kudriavzevii*, *Nakaseomyces glabratus*, *Clavispora lusitaniae*, and *Candida parapsilosis*. The integrated report summarized 24 antifungal-resistance marker hits and a median assembly N50 of 35,946 bp. The species distribution and QUAST-derived assembly-contiguity metrics for the Ugandan clinical validation dataset are summarized in Figure 3. These outputs demonstrate that rMAP-Candida can produce interpretable species typing, assembly-contiguity, AMR marker, phylogenomic, and SNP-distance summaries from a multi-species *Candida* spp. surveillance dataset. The full interactive report is available at: https://gmboowa.github.io/rMAP-Candida/reports/Ugandan_dataset/report.html.

### Assembly-contiguity summary across validation datasets

To provide a clearer overview of assembly performance across validation runs, we summarized key QUAST-derived assembly-contiguity metrics, including median N50, median number of contigs, and median total assembly length where batch-level summaries were available (Table 3). These metrics describe assembly contiguity and overall assembly size rather than genome completeness, because BUSCO and Compleasm are optional modules and may not be enabled in all rapid surveillance runs.

**TABLE 3:** Summary of assembly-contiguity statistics across rMAP-*Candida* validation datasets.

Dataset	Number of samples	Median N50 (bp)	Median number of contigs	Median total assembly length (bp)	Main interpretation
Two-sample reproducibility run	2	61,358	826	13,195,502	Minimal reproducibility test confirming expected report generation; species-aware phylogeny skipped as expected because each species had fewer than three same-species samples
Small public validation batch	17	Per-sample QUAST outputs available	Per-sample QUAST outputs available	Per-sample QUAST outputs available	Functional test and report generation check
Medium public validation batch	25	61,626	Per-sample QUAST outputs available	Per-sample QUAST outputs available	Multi-sample reporting and species-distribution assessment
Uganda clinical validation batch	34	35,946	1,529	14,708,876	Local surveillance validation using multi-species clinical *Candida* spp. reads
Large public validation batch	50	Per-sample QUAST outputs available	Per-sample QUAST outputs available	Per-sample QUAST outputs available	Scalability and report-stability assessment

As shown in Table 3, the Uganda clinical validation dataset showed a median assembly N50 of 35,946 bp, a median of 1,529 contigs, and a median total assembly length of 14,708,876 bp across 34 isolates. The two-sample reproducibility run showed a median assembly N50 of 61,358 bp and expected output generation from a fresh local Cromwell execution. Together, these dataset-level summaries complement the sample-level QUAST outputs available through the integrated report and Supplementary Table S1. A more detailed per-sample quality metrics table, including read-level QC summaries, QUAST assembly metrics, optional BUSCO or Compleasm completeness outputs where enabled, species-typing calls, antifungal-resistance marker counts, and phylogeny eligibility status, is provided as Supplementary Table S1.

### Species typing performance

Species-level assignment was generated using the custom *Candida*-focused Kraken2/Bracken database. In the representative validation report, top species calls showed high species read percentages for most isolates, supporting the utility of the species-typing module for single-isolate *Candida* spp. WGS data. The workflow also reports clade reads, taxon reads, taxonomic identifiers, and evidence source, supporting transparent review of species assignments.

### Assembly and completeness assessment

QUAST is used as the default assembly assessment module to report contig-level assembly-contiguity metrics, including total assembly length, number of contigs, largest contig, N50, and GC content. BUSCO and Compleasm are optional completeness modules and are disabled by default in the recommended rapid surveillance configuration. Therefore, QUAST-only runs should be described as assembly-contiguity assessment rather than full genome-completeness assessment.

### Antifungal-resistance marker screening

The antifungal resistance module captured marker-level evidence from ChroQueTas/FungAMR-derived outputs and presented gene/status, mutation, effect, evidence level, and interpretation fields in the final report. Positive-control behavior included detection of curated Cyp51 markers such as Y132F and V125A in selected *Candidozyma auris* validation samples. The integrated report emphasizes that genomic marker detection should be interpreted together with species identity, sample metadata, validated mutation catalogues, and phenotypic antifungal susceptibility testing where available. The multi-row parser retains concurrent mutation records as separate findings; therefore, when the scanner emits both an ERG11/Cyp51 alteration and an FCY1 mutation such as S70R in the same isolate, both are reported independently in the final AMR summary rather than one record replacing the other.

### Species-aware phylogenomics and SNP-distance reporting

rMAP-Candida includes an optional species-aware core-SNP phylogenomics module. The workflow groups samples by species and avoids combining unrelated *Candida* spp. into a single tree. For eligible species groups with sufficient samples and an available reference genome, the workflow generates core-SNP alignments, maximum-likelihood phylogenetic trees, rendered tree images, and Newick outputs. The integrated report also summarizes pairwise SNP distances and closest-neighbor relationships. These outputs are intended to support genomic epidemiology interpretation, but low SNP distances are reported conservatively as evidence of close genetic relatedness rather than direct transmission, which requires epidemiological metadata, sampling dates, and species-specific validation.

Pairwise SNP distances are reported as descriptive genomic relatedness metrics and should not be interpreted as direct evidence of transmission without epidemiological metadata, sampling dates, facility/ward information, and species-specific validation. The “possible close genetic relationship” flag is therefore intended as a conservative surveillance prompt rather than a definitive outbreak classification.

### Application to clinical *Candida spp*. isolates

The workflow was also applied to a 34-isolate Ugandan clinical validation dataset assembled from two publicly available NCBI/SRA BioProjects representing distinct clinical contexts: *Candida* spp. isolates from people living with HIV (PLWH) with oropharyngeal candidiasis attending TASO clinics in Uganda (Benson et al., 2024) and invasive catheter-associated *Candida* spp. isolates from Uganda, including candidemia-associated samples. This validation dataset enabled assessment of rMAP-Candida across clinically relevant *Candida* spp. from different specimen sources, patient groups, and study settings.

Application of rMAP-Candida to these Ugandan isolates demonstrated the workflow’s ability to generate interpretable species assignments, assembly-contiguity metrics, antifungal-resistance marker summaries, species-aware phylogenomic outputs, SNP-distance tables, and integrated surveillance reports from clinical fungal genomic data. This local validation supports the relevance of rMAP-Candida for fungal genomic surveillance in Uganda and similar settings, while providing a foundation for future analyses that link genomic outputs with clinical metadata, specimen source, epidemiological context, and phenotypic antifungal susceptibility testing. These validation datasets are summarized in Table 2.

### Runtime and resource benchmarking

Runtime and resource benchmarking was performed across small, medium, Ugandan clinical, and large validation batches to assess workflow scalability and report-generation stability. Runtime estimates varied with sample number, input read depth, enabled modules, local system load, Docker image availability, and execution environment. These benchmarking results are summarized in Table 4.

**TABLE 4:** Runtime and resource benchmarking of rMAP-*Candida*.

Dataset	Samples	Execution environment	Resource configuration	Modules enabled	Approx. runtime	Benchmarking purpose/notes
Small batch	17	Local Cromwell	MacBook Pro; 8 CPU cores; 16 GB RAM; 1 TB SSD	QC, species typing, assembly, QUAST, AMR, phylogeny	45 min	Functional test and report generation check
Medium batch	25	Local Cromwell	MacBook Pro; 8 CPU cores; 16 GB RAM; 1 TB SSD	QC, species typing, assembly, QUAST, AMR, phylogeny	2 h	Multi-sample reporting and species-distribution summary
Uganda clinical batch	34	Local Cromwell	Cloud or local resources configured according to dataset size	Full recommended surveillance mode	3 h	Local surveillance relevance using Ugandan clinical *Candida* spp. reads available from NCBI/SRA
Large batch	50	Local Cromwell	Scaled CPU, memory, and disk resources for larger batches	Full recommended surveillance mode	6 h	Scalability, sharding behaviour, and report stability

Benchmarking experiments for the rMAP-Candida workflow were conducted on a 16-inch MacBook Pro (2019) equipped with a 2.3 GHz, 8-core Intel Core i9 processor, 16 GB DDR4 RAM, and a 1 TB SSD. Runtime estimates are approximate and may vary depending on input read depth, number of samples, enabled workflow modules, Docker image availability, local system load, and whether the workflow is executed locally or on Cloud.

## Discussion

rMAP-Candida addresses a practical implementation gap in fungal genomics by integrating commonly required analytical steps for *Candida* spp. whole-genome sequencing into a single reproducible WDL/Cromwell workflow (World Health Organization, 2022). This workflow is not an introduction of a new taxonomic classifier, assembler, resistance-prediction algorithm, or phylogenetic method. Rather, it operationalizes established tools into a modular, containerized, and surveillance-oriented framework that can be executed reproducibly across local workstations, high-performance computing environments, and cloud-compatible platforms (Wratten et al., 2021; Ahmed et al., 2021; Voss et al., 2017; Merkel, 2014). This is particularly important for applied fungal genomic surveillance, where reproducibility, portability, transparent reporting, and ease of interpretation are often as important as the individual analytical tools themselves.

Several workflow management systems are widely used in bioinformatics, including WDL/Cromwell, Snakemake, Nextflow, and community frameworks such as nf-core. These systems share the broader goal of improving reproducibility, portability, and scalable execution, but they differ in syntax, ecosystem, execution backends, and community conventions. WDL/Cromwell was selected for rMAP-Candida because it provides typed workflow inputs and outputs, scatter-based parallel execution, Dockerized task runtimes, workflow metadata, and portability across local, high-performance computing, and cloud-compatible execution environments. This design is well aligned with the intended use of rMAP-Candida as a reproducible surveillance and training workflow that can be tested locally using a small dataset and then scaled to larger analyses without changing the underlying analytical logic. Nevertheless, rMAP-Candida is best viewed as one implementation of a portable fungal genomics workflow rather than as a replacement for other workflow ecosystems. Future community adoption could include equivalent wrappers or interoperability layers for Nextflow, Snakemake, or nf-core-style deployment where these systems are preferred.

rMAP-Candida is complementary to existing bioinformatics tools and workflow ecosystems rather than a replacement for them. Individual components such as fastp, Kraken2/Bracken, MEGAHIT, QUAST, BUSCO, Compleasm, Snippy, and IQ-TREE already provide robust functionality for specific analytical tasks. In addition, broader workflow frameworks and pathogen-genomics pipelines have demonstrated the value of reproducible, containerized, and community-maintained analysis systems. The contribution of rMAP-Candida is the operational integration of these concepts into a Candida-focused surveillance workflow that combines species typing, assembly-contiguity assessment, optional completeness evaluation, antifungal-resistance marker screening, species-aware phylogenomics, SNP-distance summarization, closest-neighbor reporting, and an integrated HTML report. This combination is intended to support applied fungal genomic surveillance, training, and interpretation in settings where users may need a single auditable workflow rather than a collection of disconnected command-line steps. To the best of our knowledge, no existing publicly available workflow currently integrates species typing, assembly-contiguity assessment, antifungal-resistance marker screening, and species-aware core-SNP phylogenomics into a single reproducible, Dockerized framework specifically tailored to *Candida* spp. genomic surveillance, which distinguishes rMAP-Candida from individual component tools and general-purpose pathogen-genomics pipelines.

The validation strategy demonstrates that rMAP-Candida can process *Candida* spp. whole-genome sequencing datasets of different sizes and sources. Public SRA/NCBI datasets organized into small, medium, and large validation batches supported functional testing, benchmarking, and evaluation of report generation across increasing sample numbers. The 34-isolate Ugandan clinical validation dataset provided a locally relevant use case and demonstrated that the workflow can generate interpretable outputs from a multi-species *Candida* spp. surveillance dataset. In this dataset, rMAP-Candida detected six top species groups, identified *Candida albicans* as the dominant species group, summarized assembly-contiguity metrics, reported antifungal-resistance marker hits, generated species-aware phylogenomic outputs for eligible species groups, and produced pairwise SNP-distance and closest-neighbor summaries (https://gmboowa.github.io/rMAP-Candida/reports/Ugandan_dataset/report.html).

A major strength of rMAP-Candida is its integrated reporting framework. The HTML report combines species typing, assembly metrics, antifungal-resistance marker screening, phylogeny eligibility, SNP-distance summaries, and workflow provenance in a format that can be reviewed by bioinformaticians, laboratory scientists, epidemiologists, and public health teams. This is useful because operational genomic surveillance requires more than the successful execution of command-line tools. It requires outputs that are auditable, interpretable, and sufficiently transparent to support downstream review (Ahmed et al., 2021; Boettiger, 2015). The report’s surveillance dashboard, sample-level cards, species-distribution summaries, AMR tables, phylogeny status tables, rendered trees, and downloadable outputs help users understand what was analyzed, what passed, what was skipped, and why (Figure 2).

The workflow’s conservative handling of antifungal-resistance marker outputs is another important feature. rMAP-Candida reports curated marker-level genomic evidence where detected, but it does not interpret the absence of a detected marker as phenotypic susceptibility. This distinction is essential for *Candida* spp. genomics because antifungal resistance can arise through diverse and species-specific mechanisms, including point mutations, regulatory changes, efflux-mediated resistance, copy-number variation, aneuploidy, loss of heterozygosity, and mechanisms that may not be represented in currently configured marker databases (Chaabane et al., 2019). Therefore, rMAP-Candida outputs should be interpreted as genomic screening evidence rather than definitive clinical susceptibility results. Where available, genomic findings should be reviewed alongside species identity, isolate metadata, validated mutation catalogues, local epidemiology, and phenotypic antifungal susceptibility testing (CLSI M27, 2017).

The species-aware phylogenomics design is also important. *Candida* is not a single phylogenomic unit, and combining divergent Candida-related species into one core-SNP tree would produce misleading genomic-distance interpretations. rMAP-Candida therefore groups isolates by species before variant calling, core-SNP alignment, tree inference, and SNP-distance summarization (Minh et al., 2020; Seemann, 2026). Species groups are processed only when they meet minimum sample-number requirements and have appropriate reference configuration. Groups that cannot be analyzed because of insufficient sample numbers, missing reference configuration, or failed intermediate outputs are explicitly reported as skipped rather than silently omitted. This improves transparency and helps users distinguish analytical ineligibility from biological absence of relatedness.

The current implementation of rMAP-Candida is optimized for paired-end short-read Illumina data, which remain widely used for microbial genome surveillance because of their accuracy, throughput, and compatibility with established variant-calling and assembly-quality workflows. However, long-read sequencing technologies such as Oxford Nanopore and PacBio offer important advantages for fungal genomics, including improved assembly contiguity, better resolution of repetitive regions, structural variants, copy-number variation, large insertions or deletions, and genome rearrangements. These features are particularly relevant for *Candida spp*. and related yeasts, where aneuploidy, loss of heterozygosity, genome plasticity, and structural variation can contribute to antifungal resistance and adaptation. At present, rMAP-Candida should therefore be interpreted as a short-read surveillance workflow. Future versions could incorporate long-read or hybrid assembly modules to improve genome completeness, structural-variant detection, and characterization of complex resistance-associated genomic changes.

The workflow is designed to support flexible implementation. For validated local Docker/Colima execution, the repository provides a Cromwell 91 route using the supplied example/cromwell.local.fifo_portable.conf Local/Shared File System configuration. This configuration defines task concurrency, maps WDL resource requests to Docker CPU and memory limits, uses container-visible task-script paths, and places temporary files and FIFOs under native/tmp to improve reliable execution on local machines. Dockerized tasks and explicit WDL inputs continue to support execution on workstations, HPC systems, and cloud-compatible platforms. In the default rapid surveillance configuration, QUAST provides assembly-contiguity and quality metrics such as contig count, total length, GC content, largest contig, and N50, while Compleasm or BUSCO can be enabled when gene-level completeness assessment is required. This distinction is important because QUAST-only runs should be interpreted as assembly-contiguity assessment rather than full genome-completeness assessment.

Despite these strengths, several limitations should be considered. First, antifungal-resistance interpretation depends on the completeness, curation, and versioning of the configured marker database. Second, *Candida* genome biology is complex: diploidy, heterozygosity, copy-number variation, aneuploidy, loss of heterozygosity, structural variation, and species-specific regulatory mechanisms may not be fully captured by short-read marker screening or by a single SNP-based model (Oggenfuss et al., 2025; Vande et al., 2023; Todd et al., 2019; Selmecki et al., 2010). Third, rMAP-Candida does not infer phenotypic susceptibility from absence of curated genomic markers. Fourth, species-aware trees and low SNP distances should be interpreted as genomic relatedness support rather than direct transmission evidence unless epidemiological metadata and species-appropriate analytical validation are available. Fifth, although the current release supports configurable modular execution through input JSON switches, the retained single-WDL implementation remains relatively large and may be harder to maintain than a workflow split into smaller imported task modules. Future releases will prioritize further refactoring into smaller WDL components while preserving the validated execution model and reproducibility. Finally, public SRA/ENA/BioSample metadata may be incomplete and should be verified before final surveillance interpretation. Overall, rMAP-Candida provides a practical, reproducible, modular, and extensible framework for *Candida* spp. genomic surveillance.

## Conclusion

rMAP-Candida is a Dockerized WDL/Cromwell workflow for reproducible analysis of paired-end *Candida* whole-genome sequencing data. The workflow integrates read-level quality control, *Candida*-focused species typing, *de novo* assembly, QUAST-based assembly-contiguity assessment, optional BUSCO or Compleasm genome completeness assessment, antifungal-resistance marker screening, species-aware core-SNP phylogenomics, pairwise SNP-distance summarization, closest-neighbor reporting, and integrated HTML surveillance reporting. Evaluation across public validation datasets and a 34-isolate Ugandan clinical *Candida* spp. dataset showed that rMAP-Candida can generate interpretable species assignments, assembly metrics, antifungal-resistance marker summaries, phylogenomic outputs, SNP-distance tables, closest-neighbor summaries, and reproducible integrated reports across datasets of different sizes and sources. The workflow emphasizes conservative interpretation by reporting antifungal-resistance markers as genomic screening evidence and by avoiding susceptibility claims when no curated marker is detected. Future work will strengthen validation against phenotypic antifungal susceptibility data, expand curated resistance-marker and reference-genome resources, improve database version tracking, and evaluate implementation across additional public health laboratory settings.

## Reproducibility, code availability, and workflow access

rMAP-Candida was designed as a portable, reproducible WDL/Cromwell workflow that can be executed across diverse computing environments, including local laptops and workstations, high-performance computing systems with container support, and cloud-compatible platforms. The workflow uses Dockerized tasks to standardize software dependencies, minimize environment-specific installation barriers, and improve consistency across users and sites. This design enables reproducible execution for training, benchmarking, method validation, and applied fungal genomic surveillance in both resource-limited and well-resourced settings. The rMAP-Candida source code, workflow file, example inputs, Docker/database documentation, the AMR container build recipe, the container validation test, validation scripts, and quick-start instructions are available from the project repository: https://github.com/gmboowa/rMAP-Candida. Example workflow reports and rendered HTML outputs are available through the project reporting page: https://gmboowa.github.io/rMAP-Candida/.

## Data Availability

The public *Candida* spp. sequencing datasets used for workflow validation were obtained from publicly available NCBI/SRA resources. The Ugandan clinical *Candida* spp. validation dataset comprised de-identified genomic sequence data from two publicly available NCBI BioProjects: PRJNA1168696, representing *Candida* species raw sequence reads from people living with HIV with oropharyngeal candidiasis attending TASO clinics in Uganda, and PRJNA1258412, representing invasive catheter-associated *Candida* species from Uganda. Accession numbers, example inputs, workflow configuration files, and representative output reports are provided through the rMAP-Candida repository and project reporting page where applicable. The representative integrated HTML report is available at: https://gmboowa.github.io/rMAP-Candida/reports/Ugandan_dataset/report.html. Per-sample quality metrics, including detailed QUAST, BUSCO/Compleasm, species-typing, and antifungal-resistance marker outputs, are provided as Supplementary Table S1. The rMAP-Candida workflow is available at https://github.com/gmboowa/rMAP-Candida. The repository includes the main WDL workflow, example input JSON files, a two-sample reproducibility test, documentation, Docker/database access checks, the AMR container Dockerfile under docker/amr_chroquetas/Dockerfile, example/cromwell.local.fifo_portable.conf, the container validation test under docker/amr_chroquetas/test/container_validation_test.sh, validation scripts, and example reports.
